# Supplementary material for: The Effectiveness of Zinc-Biofortified Wheat Flour Intake on the Growth and Morbidity Outcomes of Rural Pakistani Children and Adolescent Girls: A Cluster-Randomised, Double-Blind, Controlled Trial
Source: Nutrients. 2025 Mar 25;17(7):1137. doi: 10.3390/nu17071137 (PMC11990903; doi:10.3390/nu17071137)
Supplement: Supplementary file 1 [file nutrients-17-01137-s001.zip › nutrients-3530980-supplementary.pdf]

Table S1. Incidence of respiratory tract infection in adolescent females by study arm.

Time point	Incidence	N total	Control		Intervention		X^2	*p*
			N	n (%)	N	n (%)		
**Baseline**								
Round 1 (week 2)		396	208		188		2.118	0.146
	Yes			19 (9.1)		10 (5.3)		
	No			189 (90.9)		178 (94.7)		
Round 2 (week 4)		397	210		187		1.793	0.181
	Yes			32 (15.2)		20 (10.7)		
	No			178 (84.8)		167 (89.3)		
Round 3 (week 6)		390	211		179		0.175	0.676
	Yes			40 (19.0)		31 (17.3)		
	No			171 (81.0)		148 (82.7)		
Round 4 (week 8)		405	207		198		0.004	0.948
	Yes			35 (16.9)		33 (16.7)		
	No			172 (83.1)		165 (83.3)		
**Mid-point**								
Round 5 (week 10)		399	212		187		0.058	0.810
	Yes			44 (20.8)		37 (19.8)		
	No			168 (79.2)		150 (80.2)		
Round 6 (week 12)		402	211		191		0.026	0.871
	Yes			40 (19.0)		35 (18.3)		
	No			171 (81.0)		156 (81.70)		
Round 7 (week 14)		411	215		196		0.233	0.629
	Yes			59 (27.4)		58 (29.6)		
	No			156 (72.6)		138 (70.4)		
Round 8 (week 16)		403	212		191		0.184	0.668
	Yes			29 (13.7)		29 (15.2)		
	No			183 (86.3)		162 (84.8)		
Round 9 (week 18)		396	210		186		0.225	0.635
	Yes			40 (19.0)		32 (17.2)		
	No			170 (81.0)		154 (82.8)		
**Endpoint**								
Round 10 (week 20)		384	197		187		1.871	0.171
	Yes			40 (20.3)		28 (15.0)		
	No			157 (79.7)		159 (85.0)		
Round 11 (week 22)		375	192		183		4.355	0.037
	Yes			37 (19.3)		21 (11.5)		
	No			155 (80.7)		162 (88.5)		
Round 12 (week 24)		380	208		172		0.094	0.759
	Yes			35 (16.8)		31 (18.0)		
	No			173 (83.2)		141 (82.0)		
Round 13 (week 26)		327	179		148		6.044	0.014
	Yes			26 (14.5)		9 (6.10)		
	No			153 (85.5)		139 (93.9)		
Round 14 (week 28)		394	210		184		0.040	0.841
	Yes			23 (11.0)		19 (10.3)		
	No			187 (89.0)		165 (89.7)		

X^2, chi-squared test values. p-values based on Pearson's chi-squared test. Significance was set at $p < 0.05$. Morbidity data was collected every two weeks covering a total period of 28 weeks through 14 rounds of data collection. Data collected in rounds 1-4 was considered as baseline period, rounds 5-9 as mid-point, and rounds 10-14 as endline period.

Table S2. Incidence of respiratory tract infection in children by study arm.

Time point	Incidence	N total	Control		Intervention		X^2	*p*
			N	n (%)	N	n (%)		
**Baseline**								
Round 1 (week 2)		395	207		188		0.030	0.863
	Yes			31 (15.0)		27 (14.4)		
	No			176 (85.0)		161(85.6)		
Round 2 (week 4)		397	210		187		3.402	0.065
	Yes			32 (15.2)		42 (22.5)		
	No			178 (84.8)		145 (77.5)		
Round 3 (week 6)		389	211		178		0.188	0.664
	Yes			39 (18.5)		36 (20.2)		
	No			172 (81.5)		142 (79.8)		
Round 4 (week 8)		406	208		198		0.562	0.454
	Yes			53 (25.5)		57 (28.8)		
	No			155 (74.5)		141 (71.2)		
**Mid-point**								
Round 5 (week 10)		411	215		196		0.067	0.796
	Yes			75 (34.9)		66 (33.7)		
	No			140 (65.1)		130 (66.3)		
Round 6 (week 12)		402	211		191		0.381	0.537
	Yes			69(32.7)		57 (29.8)		
	No			142(67.3)		134 (70.2)		
Round 7 (week 14)		411	215		196		.067	0.796
	Yes			75(34.9)		66 (33.7)		
	No			140(65.1)		130 (66.3)		
Round 8 (week 16)		403	212		191		3.196	0.074
	Yes			62 (29.2)		41 (21.5)		
	No			150 (70.8)		150 (78.5)		
Round 9 (week 18)		396	210		186		0.222	0.637
	Yes			69 (32.9)		57 (30.6)		
	No			141 (67.1)		129 (69.4)		
**Endpoint**								
Round 10 (week 20)		384	197		187		.000	0.995
	Yes			58 (29.4)		55 (29.4)		
	No			139 (70.6)		132 (70.6)		
Round 11 (week 22)		373	192		181		1.632	0.201
	Yes			58 (30.2)		44 (24.3)		
	No			134 (69.8)		137 (75.7)		
Round 12 (week 24)		380	208		172		1.412	0.235
	Yes			66 (31.7)		45 (26.2)		
	No			142 (68.3)		127 (73.8)		
Round 13 (week 26)		327	179		148		4.408	0.036
	Yes			49(27.4)		26 (17.6)		
	No			130(72.6)		122 (82.4)		
Round 14 (week 28)		394	210		184		1.495	0.222
	Yes			47(22.4)		51 (27.7)		
	No			163 (77.6)		133 (72.3)		

X^2, chi-squared test values. p-values based on Pearson's chi-squared test. Significance was set at $p < 0.05$. Morbidity data was collected every two weeks covering a total period of 28 weeks through 14 rounds of data collection. Data collected in rounds 1-4 was considered as baseline period, rounds 5-9 as mid-point, and rounds 10-14 as endline period.

Table S3. Duration of respiratory tract infection (in days) for the adolescent girls by study arm.

Time point	n	Control	n	Intervention	β (95%CI) *	t	*p*
**Baseline:**							
Round 1 (week 2)	208	0.79 ± 2.78	188	0.30 ± 1.47	-0.665 (-1.290, -0.041)	-2.150	0.037
Round 2 (week 4)	210	1.38 ± 3.50	187	0.75 ± 2.47	-0.995 (-1.983, -0.006)	-2.036	0.049
Round 3 (week 6)	211	1.37 ± 3.22	179	1.03 ± 2.66	-0.909 (-1.865, 0.046)	-1.932	0.061
Round 4 (week 8)	207	1.17 ± 3.08	198	1.04 ± 2.64	-0.224 (-0.967,0.519)	-0.615	0.543
**Mid-point:**							
Round 5 (week 10)	211	1.34 ± 3.03	187	1.05 ± 2.47	-0.456 (-1.208, 0.296)	-1.228	0.227
Round 6 (week 12)	211	1.52 ± 3.44	191	1.48 ±3.56	-0.271 (-1.283, 0.741)	-0.541	0.592
Round 7 (week 14)	215	2.23 ± 4.20	196	2.19 ± 3.91	-0.246 (-1.557, 1.066)	-0.376	0.708
Round 8 (week 16)	212	1.12 ± 3.22	191	1.13 ± 3.09	0.062 (-0.924, 1.048)	0.127	0.900
Round 9 (week 18)	210	1.77 ± 3.95	186	1.45 ± 3.56	-1.266 (-2.614, 0.083)	-1.882	0.065
**Endline:**							
Round 10 (week 20)	197	1.51 ± 3.50	187	0.95 ± 2.69	-0.528 (-1.468, 0.412)	-1.131	0.264
Round 11 (week 22)	192	1.38 ± 3.29	183	0.89 ± 2.80	-0.962 (-2.124,0.200)	-1.670	0.102
Round 12 (week 24)	208	1.38 ± 3.52	172	1.25 ± 3.14	-0.189 (-1.066,0.688)	-0.442	0.662
Round 13 (week 26)	179	1.10 ± 3.12	148	0.60 ± 2.50	-0.668 (-1.638, 0.302)	-1.415	0.169
Round 14 (week 28)	210	0.73 ± 2.46	184	0.67 ± 2.32	-0.053 (-0.647, 0.542)	-0.181	0.858

Data presented as mean ± SD. *Values represent beta coefficient and 95% CI from linear regression models. p-values obtained using linear mixed models adjusted for cluster effect to test differences between the groups for continuous variables. Significance was set at $p < 0.05$. Morbidity data was collected every two weeks covering a total period of 28 weeks through 14 rounds of data collection. Data collected in rounds 1-4 was considered as baseline period, rounds 5-9 as mid-point, and rounds 10-14 as endline period.

Table S4. Duration of respiratory tract infection (in days) for the children by study arm.

Time Points	n	Control	n	Intervention	β (95%CI) *	t	*p*
**Baseline:**							
Round 1 (week 2)	206	1.33 ± 3.51	188	1.07 ± 2.97	-0.449 (-1.354,0.457)	-1.002	0.323
Round 2 (week 4)	210	0.92 ± 2.52	187	1.28 ± 2.73	0.312 (-0.440, 1.065)	0.842	0.405
Round 3 (week 6)	211	1.18 ± 2.88	178	1.16 ± 2.65	-0.180 (-1.016, 0.656)	-0.437	0.665
Round 4 (week 8)	208	1.57 ± 3.28	198	1.84 ± 3.42	0.151 (-0.793, 1.095)	0.325	0.747
**Mid-point:**							
Round 5 (week 10)	211	2.33 ± 3.68	187	2.21 ± 3.50	-0.124 (-1.170, 0.923)	-0.239	0.813
Round 6 (week 12)	211	2.46 ± 4.01	191	2.06 ± 3.79	-1.126 (-2.591,0.339)	-1.536	0.130
Round 7 (week 14)	215	2.85 ± 4.33	196	2.47 ± 4.13	-0.684 (-2.199, 0.831)	-0.903	0.370
Round 8 (week 16)	212	2.10 ± 3.87	191	1.67 ± 3.69	-0.693(-1.973, 0.586)	-1.087	0.282
Round 9 (week 18)	210	2.47 ± 4.13	186	2.57 ± 4.40	-0.922 (-2.491,0.648)	-1.173	0.245
**Endline:**							
Round 10 (week 20)	197	2.18 ± 4.06	187	1.96 ± 3.58	-0.326 (-1.591, 0.939)	-0.517	0.607
Round 11 (week 22)	192	2.09 ± 3.93	181	1.49 ± 3.32	-1.328 (-2.786, 0.130)	-1.834	0.073
Round 12 (week 24)	208	2.34 ± 4.14	172	1.80 ± 3.59	-1.090 (-2.58, 0.402)	-1.463	0.149
Round 13 (week 26)	179	2.17 ± 4.15	148	1.31 ± 3.32	-0.261 (-1.927, 1.404)	-0.315	0.754
Round 14 (week 28)	210	1.64 ± 3.59	184	1.97 ± 3.81	0.367 (-0.852, 1.586)	0.606	0.547

Data presented as mean ± SD. *Values represent beta coefficient and 95% CI from linear regression models. p-values obtained using linear mixed models adjusted for cluster effect to test differences between the groups for continuous variables. Significance was set at $p < 0.05$. Morbidity data was collected every two weeks covering a total period of 28 weeks through 14 rounds of data collection. Data collected in rounds 1-4 was considered as baseline period, rounds 5-9 as mid-point, and rounds 10-14 as endline period.

Table S5. Duration of respiratory tract infections (in days) among only sick adolescent girls by study arm.

Time Points	n	Control	n	Intervention	β (95%CI) *	t	*p*
**Baseline:**							
Round 1 (week 2)	19	8.63 ± 4.17	10	5.70 ± 3.30	-2.932 (-6.05, 0.194)	-1.924	0.065
Round 2 (week 4)	32	9.06 ± 3.28	20	7.05 ± 3.57	-1.855 (-5.010, 1.309)	-1.304	0.221
Round 3 (week 6)	40	7.23 ± 3.55	31	5.94 ± 3.46	-1.166 (-3.490, 1.159)	-1.049	0.307
Round 4 (week 8)	35	6.94 ± 4.03	33	6.21 ± 3.11	-1.099 (-3.599, 1.401)	-0.913	0.371
**Mid-point:**							
Round 5 (week 10)	44	6.55 ± 3.27	37	5.30 ± 2.89	-1.314 (-3.174, 0.545)	-1.463	0.157
Round 6 (week 12)	40	8.00 ± 3.23	35	8.06 ± 4.04	-0.090 (-1.909, 1.728)	-0.102	0.919
Round 7 (week 14)	59	8.12 ± 4.06	58	7.40 ± 3.62	-0.892 (-2.915, 1.132)	-0.905	0.374
Round 8 (week 16)	29	8.21 ± 4.25	29	7.45 ± 4.02	-0.687 (-3.066, 1.693)	-0.600	0.555
Round 9 (week 18)	40	9.30 ± 3.43	32	8.41 ± 3.92	-1.641 (-3.890, 0.609)	-1.493	0.146
**Endline:**							
Round 10 (week 20)	40	7.43 ± 4.06	28	6.36 ± 3.79	-1.084 (-3.343, 1.175)	-1.012	0.326
Round 11 (week 22)	37	7.16 ± 3.85	21	7.71 ± 3.98	0.120 (-2.477, 2.717)	0.096	0.924
Round 12 (week 24)	35	8.23 ± 4.19	31	6.94 ± 3.94	-1.241 (-3.609, 1.126)	-1.114	0.282
Round 13 (week 26)	26	7.58 ± 4.28	9	9.89 ± 3.33	2.334 (-1.595, 6.264)	1.255	0.227
Round 14 (week 28)	23	6.70 ± 3.98	19	6.53 ± 3.78	-0.162 (-3.693, 3.369)	-0.097	0.924

Data presented as mean ± SD. *Values represent beta coefficient and 95% CI from linear regression models. p-values obtained using linear mixed models adjusted for cluster effect to test differences between the groups for continuous variables. Significance was set at $p < 0.05$. Morbidity data was collected every two weeks covering a total period of 28 weeks through 14 rounds of data collection. Data collected in rounds 1-4 was considered as baseline period, rounds 5-9 as mid-point, and rounds 10-14 as endline period.

Table S6. Duration of respiratory tract infections (in days) among only sick children by study arm.

**Time Points**	**n**	**Control**	**n**	**Intervention**	**β (95%CI) ***	**t**	***p***
**Baseline:**							
Round 1 (week 2)	30	9.10 ± 3.70	27	7.48 ± 3.67	-1.619 (-3.578, 0.341)	-1.655	0.104
Round 2 (week 4)	32	6.06 ± 3.28	42	5.69 ± 2.86	-0.095 (-1.954, 1.764)	-0.109	0.915
Round 3 (week 6)	39	6.41 ± 3.39	36	5.75 ± 2.91	-0.660(-2.121, 0.801)	-0.901	0.371
Round 4 (week 8)	53	6.15 ± 3.75	57	6.40 ± 3.36	0.685 (-1.235, 2.605)	0.734	0.469
**Mid-point:**							
Round 5 (week 10)	76	6.47 ± 3.28	64	6.45 ± 2.87	0.127 (-1.324, 1.579)	0.179	0.859
Round 6 (week 12)	69	7.52 ± 3.31	57	6.91 ± 3.82	-0.861 (-2.415, 0.693)	-1.144	0.264
Round 7 (week 14)	75	8.17 ± 3.19	66	7.33 ± 3.85	-0.840 (-2.013, 0.333)	-1.416	0.159
Round 8 (week 16)	62	7.19 ± 3.83	41	7.78 ± 4.01	0.685 (-1.467, 2.837)	0.646	0.523
Round 9 (week 18)	69	7.51 ± 3.73	57	8.39 ± 3.80	1.109 (-0.971,3.189)	1.086	0.286
**Endline:**							
Round 10 (week 20)	58	7.40 ± 4.18	55	6.65 ± 3.51	-0.788 (-2.605,1029)	-0.903	0.377
Round 11 (week 22)	58	6.91 ± 4.23	44	6.11 ± 4.15	-0.778 (-2.949, 1.393)	-0.730	0.471
Round 12 (week 24)	66	7.36 ± 4.13	45	6.89 ± 3.79	-0.229 (-2.450,1.992)	-0.210	0.835
Round 13 (week 26)	49	7.92 ± 4.17	26	7.46 ± 4.12	0.107 (-2.244, 2.459)	0.092	0.927
Round 14 (week 28)	47	7.32 ± 4.03	51	7.12 ± 3.98	-0.187(-2.233, 1.859)	-0.186	0.854

Data presented as mean ± SD. *Values represent beta coefficient and 95% CI from linear regression models. p-values obtained using linear mixed models adjusted for cluster effect to test differences between the groups for continuous variables. Significance was set at $p < 0.05$. Morbidity data was collected every two weeks covering a total period of 28 weeks through 14 rounds of data collection. Data collected in rounds 1-4 was considered as baseline period, rounds 5-9 as mid-point, and rounds 10-14 as endline period.

Table S7. Incidence of diarrhoea in the participating children by study arm.

Time point	Incidence	N total	Control		Intervention		X^2	*p*
			n	n (%)	n	n (%)		
**Baseline:**								
Round 1 (week 2)		397	208		189		2.166	0.141
	Yes			35 (16.8)		22 (11.6)		
	No			173 (83.2)		167 (88.4)		
Round 2 (week 4)		395	209		186		0.005	0.943
	Yes			32 (15.3)		28 (15.1)		
	No			177 (84.7)		158 (84.9)		
Round 3 (week 6)		388	211		177		1.912	0.167
	Yes			45 (21.3)		28 (15.8)		
	No			166 (78.7)		149 (84.2)		
Round 4 (week 8)		405	207		198		0.950	0.330
	Yes			36 (17.4)		42 (21.2)		
	No			171 (82.6)		156 (78.8)		
**Mid-point:**								
Round 5 (week 10)		399	212		187		1.223	0.269
	Yes			33 (15.6)		37 (19.8)		
	No			179 (84.4)		150 (80.2)		
Round 6 (week 12)		402	211		191		1.650	0.199
	Yes			22 (10.4)		28 (14.7)		
	No			189 (89.6)		163 (85.3)		
Round 7 (week 14)		411	215		196		0.401	0.526
	Yes			25 (11.6)		19 (9.7)		
	No			190 (88.4)		177 (90.3)		
Round 8 (week 16)		403	212		191		0.102	0.749
	Yes			22 (10.4)		18 (9.4)		
	No			190 (89.6)		173 (90.6)		
Round 9 (week 18)		396	210		186		0.146	0.702
	Yes			18 (8.6)		18 (9.7)		
	No			192 (91.4)		168 (90.3)		
**Endpoint:**								
Round 10 (week 20)		384	197		187		1.276	0.259
	Yes			25 (12.7)		17 (9.1)		
	No			172 (87.3)		170 (90.9)		
Round 11 (week 22)		375	192		183		1.788	0.181
				20 (10.4)		12 (6.6)		
	Yes			172 (89.6)		171 (93.4)		
	No							
Round 12 (week 24)		380	208		172		0.820	0.365
	Yes			11 (5.3)		13 (7.6)		
	No			197 (94.7)		159 (92.4)		
Round 14 (week 28)		327	179		148		1.288	0.256
	Yes			14 (7.8)		7 (4.7)		
	No			165 (92.2)		141 (95.3)		
		394	210		184		0.082	0.774
	Yes			9 (4.3)		9 (4.9)		
	No			201 (95.7)		175 (95.1)		

X^2, chi-squared test values. p-values based on Pearson's chi-squared test. Significance was set at $p < 0.05$. Morbidity data was collected every two weeks covering a total period of 28 weeks through 14 rounds of data collection. Data collected in rounds 1-4 was considered as baseline period, rounds 5-9 as mid-point, and rounds 10-14 as endline period.

Table S8. Duration of diarrhoea (in days) for the children by study arm.

Time Points	n	Control	n	Intervention	β (95%CI) *	t	*p*
**Baseline:**							
Round 1 (week 2)	208	0.75 ± 1.98	189	0.58 ± 1.89	-0.317 (-0.939, 0.305)	-1.025	0.311
Round 2 (week 4)	209	0.67 ± 1.78	186	0.59 ± 1.58	-0.369 (-0.922, 0.185)	-1.351	0.185
Round 3 (week 6)	211	0.95 ± 2.18	177	0.75 ± 2.02	-0.304 (-0.985, 0.377)	-0.905	0.372
Round 4 (week 8)	207	0.94 ± 2.40	198	0.97 ± 2.15	-0.258 (-1.038, 0.522)	-0.666	0.509
**Mid-point:**							
Round 5 (week 10)	212	0.79 ± 2.09	187	1.00 ± 2.37	0.021 (-0.655, 0.696)	0.062	0.951
Round 6 (week 12)	211	0.53 ± 1.79	191	0.84 ± 2.40	0.185 (-0.544, 0.914)	0.513	0.611
Round 7 (week 14)	215	0.70 ± 2.27	196	0.58 ± 2.13	-0.237 (-0.866, 0.392)	-0.762	0.450
Round 8 (week 16)	212	0.78 ± 2.60	191	0.53 ± 1.90	-0.39 (-1.108, 0.327)	-1.093	0.280
Round 9 (week 18)	210	0.62 ± 2.38	186	0.60 ± 2.15	-0.142 (-0.845, 0.562)	-0.406	0.687
**Endline:**							
Round 10 (week 20)	197	0.81 ± 2.46	187	0.43 ± 1.62	-0.488 (-1.121, 0.145)	-1.553	0.128
Round 11 (week 22)	192	0.62 ± 2.23	183	0.45 ± 2.02	-0.314 (-0.932, 0.303)	-0.932	0.303
Round 12 (week 24)	208	0.33 ± 1.72	172	0.47 ± 2.03	0.148 (-0.381, 0.676)	0.569	0.573
Round 13 (week 26)	179	0.40 ± 1.65	148	0.32 ± 1.65	-0.295 (-0.872, 0.282)	-1.044	0.305
Round 14 (week 28)	210	0.20 ± 1.18	184	0.39 ± 1.91	0.125 (-0.287, 0.536)	0.634	0.533

Data presented as mean ± SD. *Values represent beta coefficient and 95% CI from linear regression models. p-values obtained using linear mixed models adjusted for cluster effect to test differences between the groups for continuous variables. Significance was set at $p < 0.05$. Morbidity data was collected every two weeks covering a total period of 28 weeks through 14 rounds of data collection. Data collected in rounds 1-4 was considered as baseline period, rounds 5-9 as mid-point, and rounds 10-14 as endline period.

Table S9. Duration of diarrhoea (in days) among only sick children by study arm.

Time Points	n	Control	n	Intervention	β (95%CI) *	t	*p*
**Baseline:**							
Round 1 (week 2)	35	4.43 ± 2.66	22	5.00 ± 2.96	0.571 (-0.944, 2.086)	0.756	0.453
Round 2 (week 4)	32	4.34 ± 2.21	28	3.93 ± 1.88	-0.415 (-1.484,0.654)	-0.777	0.440
Round 3 (week 6)	45	4.44 ± 2.59	28	4.75 ± 2.65	0.306 (-0.948, 1.559)	0.486	0.628
Round 4 (week 8)	36	5.39 ± 3.05	42	4.57 ± 2.32	-0.894 (-2.305, 0.516)	-1.318	0.202
**Mid-point:**							
Round 5 (week 10)	33	5.09 ± 2.50	37	5.05 ± 2.84	-0.037 (-1.320, 1.247)	-0.057	0.954
Round 6 (week 12)	22	5.05 ± 2.87	28	5.75 ± 3.35	0.705 (-1.100, 2.509)	0.785	0.436
Round 7 (week 14)	25	6.04 ± 3.53	19	6.00 ± 3.86	0.041(-2.556, 2.638)	0.034	0.973
Round 8 (week 16)	22	7.50 ± 3.88	18	5.67 ± 3.09	-1.520 (-4.912, 1.729)	-1.052	0.315
Round 9 (week 18)	18	7.28 ± 4.25	18	6.17 ± 3.71	-1.111 (-3.816, 1.594)	-0.835	0.410
**Endline:**							
Round 10 (week 20)	25	6.40 ± 3.48	17	4.71 ± 3.04	-1.835 (-4.558, 0.888)	-1.435	0.172
Round 11 (week 22)	20	5.95 ± 4.07	12	6.83 ± 4.47	1.283 (-3.013, 5.579)	0.660	0.523
Round 12 (week 24)	11	6.27 ± 4.50	13	6.23 ± 4.46	-0.042 (-3.843, 3.760)	-0.023	0.982
Round 13 (week 26)	14	5.07 ± 3.45	7	6.86 ± 3.80	1.696 (-2.594, 5.985)	0.889	0.396
Round 14 (week 28)	9	4.67 ± 3.61	9	7.89 ± 4.11	3.203 (-1.901, 8.307)	1.513	0.178

Data presented as mean ± SD. *Values represent beta coefficient and 95% CI from linear regression models. p-values obtained using linear mixed models adjusted for cluster effect to test differences between the groups for continuous variables. Significance was set at $p < 0.05$. Morbidity data was collected every two weeks covering a total period of 28 weeks through 14 rounds of data collection. Data collected in rounds 1-4 was considered as baseline period, rounds 5-9 as mid-point, and rounds 10-14 as endline period.
